# Linoleic and palmitoleic acid block streptokinase-mediated plasminogen activation and reduce severity of invasive group A streptococcal infection

**DOI:** 10.1038/s41598-017-11276-z

**Published:** 2017-09-18

**Authors:** Katharina Rox, Rolf Jansen, Torsten G. Loof, Christine M. Gillen, Steffen Bernecker, Mark J. Walker, Gursharan Singh Chhatwal, Rolf Müller

**Affiliations:** 1 Department of Microbial Natural Products, Helmholtz Institute for Pharmaceutical Research (HIPS), Helmholtz Centre for Infection Research (HZI), Saarland University, Saarbrücken, Germany; 2 Department of Medical Microbiology, Helmholtz Centre for Infection Research (HZI), Braunschweig, Germany; 3 Central facility for Microscopy, Helmholtz Centre for Infection Research (HZI), Braunschweig, Germany; 4 Department of Microbial Drugs, Helmholtz Centre for Infection Research (HZI), Braunschweig, Germany; 5 Infection Immunology Research Group, Helmholtz Centre for Infection Research (HZI), Braunschweig, Germany; 6 German Centre for Infection Research (DZIF), Partner Site Braunschweig-Hannover, Hannover, Germany; 7 School of Chemistry and Molecular Biosciences and Australian Infectious Disease Research Centre, The University of Queensland, St. Lucia, Queensland Australia

## Abstract

In contrast to mild infections of Group A *Streptococcus* (GAS) invasive infections of GAS still pose a serious health hazard: GAS disseminates from sterile sites into the blood stream or deep tissues and causes sepsis or necrotizing fasciitis. In this case antibiotics do not provide an effective cure as the bacteria are capable to hide from them very quickly. Therefore, new remedies are urgently needed. Starting from a myxobacterial natural products screening campaign, we identified two fatty acids isolated from myxobacteria, linoleic and palmitoleic acid, specifically blocking *streptokinase-mediated* activation of plasminogen and thereby preventing streptococci from hijacking the host’s plasminogen/plasmin system. This activity is not inherited by other fatty acids such as oleic acid and is not attributable to the killing of streptococci. Moreover, both fatty acids are superior in their inhibitory properties compared to two clinically used drugs (tranexamic or ε-amino caproic acid) as they show 500–1000 fold lower IC_50_ values. Using a humanized plasminogen mouse model mimicking the clinical situation of a local GAS infection that becomes systemic, we demonstrate that these fatty acids ameliorate invasive GAS infection significantly. Consequently, linoleic and palmitoleic acid are possible new options to combat GAS invasive diseases.

## Introduction

Every year millions of people suffer from group A streptococcal (GAS) diseases ranging from mild infections to severe and life-threatening syndromes including sepsis and necrotizing fasciitis. The latter are designated invasive diseases as bacteria are isolated from usually sterile sites such as deep tissues or the blood stream^1^. It is estimated that over 660,000 cases of invasive Group A *Streptococcus* (GAS) infections and over 160,000 deaths occur each year^2^. Even under treatment GAS invasive infections exhibit a high mortality rate of about 15–20%^3^. As a vaccine is not commercially available yet^4,5^, new drugs are urgently needed to successfully combat GAS invasive infections.

GAS hijack the host factor plasminogen during invasive diseases^6,7^ by secreting streptokinase, a specific human plasminogen activator. Streptokinase activates plasminogen to plasmin, allowing GAS to disseminate into deeper tissue^8^ or lyse fibrin clots in which they may be entrapped^9,10^. Streptokinase is a single-chain, 414-amino-acid protein which is composed of three different domains: an α-, β- and a γ-domain^11^. Streptokinase can be classified into three so-called ‘cluster types’. Cluster 1 type streptokinase is secreted by streptococci and forms a complex with plasminogen directly, triggering a conformational change in the plasminogen molecule which then cleaves the Arg_561_-Val_562_ bond of another plasminogen molecule activating it to plasmin. Cluster 2 type streptokinase needs fibrinogen for activation of plasminogen. Cluster 2a type streptokinase is secreted and forms a tri-molecular complex with fibrinogen and plasminogen to activate plasminogen to plasmin. Cluster 2b type streptokinase is only able to activate plasminogen on the bacterial cell surface; plasminogen is bound to the streptococcal cell surface via plasminogen-binding group A streptococcal M or M-like protein. Then, a tri-molecular complex is formed (fibrinogen-plasminogen-streptokinase) activating further plasminogen molecules^12,13^. Additionally, it has been shown that cluster 2a type streptokinase can activate plasminogen in the absence of fibrinogen although it does not act as fast as cluster 1 type streptokinase^14^. Streptokinase can also form a complex with plasmin. This complex activates plasminogen more rapidly than a streptokinase-plasminogen-complex^15^. All three cluster types activate soluble plasminogen when formed into a streptokinase-plasmin-complex^16^. The 92 kDa single-chain plasminogen is a glycoprotein consisting of 791 amino acids^17^. A small molecule inhibitor directed against streptokinase has not been described. However, inhibitors of streptokinase gene expression have shown promise for the development of potential therapeutics^18,19^.

Here, we identify two fatty acids isolated from myxobacteria, linoleic and palmitoleic acid, which block *streptokinase-mediated* activation of plasminogen. Using a humanized plasminogen mouse model which mimics a local group A streptococcal infection that becomes systemic, we demonstrate that these fatty acids ameliorate invasive GAS infection. Thereby, we provide evidence supporting the concept that these fatty acids can act as anti-virulence agents against GAS invasive infection. Consequently, linoleic and palmitoleic acid are possible new options for the treatment of invasive GAS disease.

## Results

### Natural products screening campaign reveals promising inhibitors of streptokinase-mediated plasminogen activation

About 600 myxobacterial extracts and 300 myxobacterial compounds from our internal library were screened for their capacity of inhibition of the activation of plasminogen by streptokinase using well established assays to measure plasminogen activation by streptokinase^13,14,16^. Several myxobacterial extracts showed high inhibitory activity and reduced the generation of plasmin dramatically. To determine which peak in the chromatogram was responsible for activation, HPLC-fractionation was performed, revealing two peaks in the chromatogram responsible for the inhibitory activity in the plasminogen activation assay (Fig. S1a,b). For isolation of the two compounds giving the activity in the chromatogram, the strain 706^20^ was selected as it yielded the highest inhibitory activity compared to equal amounts of other myxobacterial strains. To assure a high yield of both compounds, the strain 706 was optimized with respect to production of both compounds by testing different media and harvesting time points. The optimal harvesting time point and the optimal medium were selected due to the activity in the facilitated plasminogen activation assay. After fermentation of the strain 706 the compounds (RC 36.1 and RC 36.2) were isolated by activity-guided fractionation (Fig. S1c,d) using different LC-MS fractionations that were tested in the facilitated plasminogen activation assay.

### Structure elucidation of RC 36.1 and RC 36.2 reveals them as palmitoleic and linoleic acid

**Figure 1:**
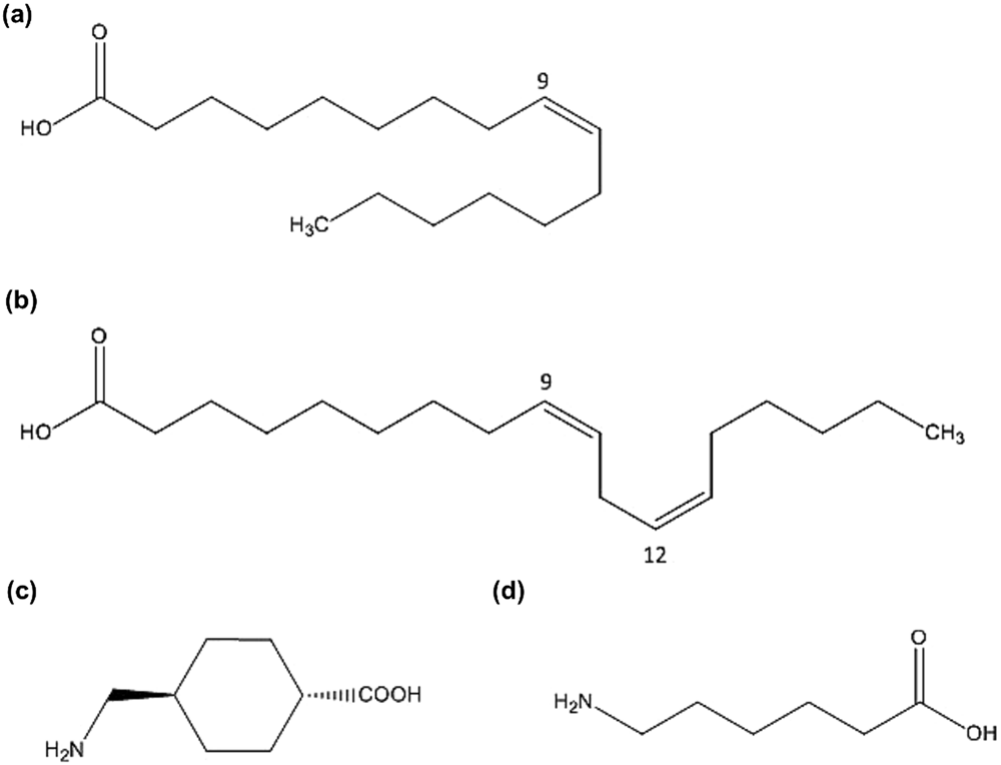
Molecular structures of palmitoleic acid, linoleic acid, tranexamic acid and ε-amino caproic acid. The molecular structures of (**a**) palmitoleic acid (PA), (**b**) linoleic acid (LA), (**c**) tranexamic acid (TXA) and (**d**) ε-amino caproic acid (EACA) are displayed.

To elucidate the structure of RC 36.1 and RC 36.2, HR-ESIMS and ^1^H- and ^13^C-NMR spectra were recorded. According to the HR-ESIMS, RC 36.1 had the elemental formula C_16_H_30_O_2_ ([M + H]^+^ *m/z* 255.2327; calcd. 255.2319) whereas RC 36.2 was C_18_H_32_O_2_ ([M + H]^+^ *m/z* 281.2477; calcd. 281.2475). The NMR spectra revealed that RC 36.1 was a mono-unsaturated C_16_-carboxylic acid whereas RC 36.2 was a double-unsaturated C_18_-carboxylic acid (Figs S2–S5, Tables S1 and S2). As NMR data did not indicate the exact position of the double bond(s), derivatization of both fatty acids (RC 36.1 and RC 36.2) to DMOX (dimethyl oxazoline) derivatives was performed. This method allows to specifically locate double bonds within fatty-acids^21^. As a result, the position of double bond(s) was successfully located for RC 36.1 and RC 36.2 at position 9 and 9,12, respectively (Fig. S6). To determine the exact conformation of the double bonds, NMR spectra of RC 36.1 were compared to the reference compounds palmitoleic (*Z*) and palmitelaidic (*E*) acid whereas NMR spectra of RC 36.2 were compared to linoleic (*Z,Z*) and linolelaidic (*E,E*) acid. RC 36.1 was identical to palmitoleic acid (PA) and RC 36.2 was identical to linoleic acid (LA) (Fig. 1a,b; Figs S2–S5).

### Linoleic and palmitoleic acid specifically inhibit streptokinase-mediated plasminogen activation

**Figure 2:**
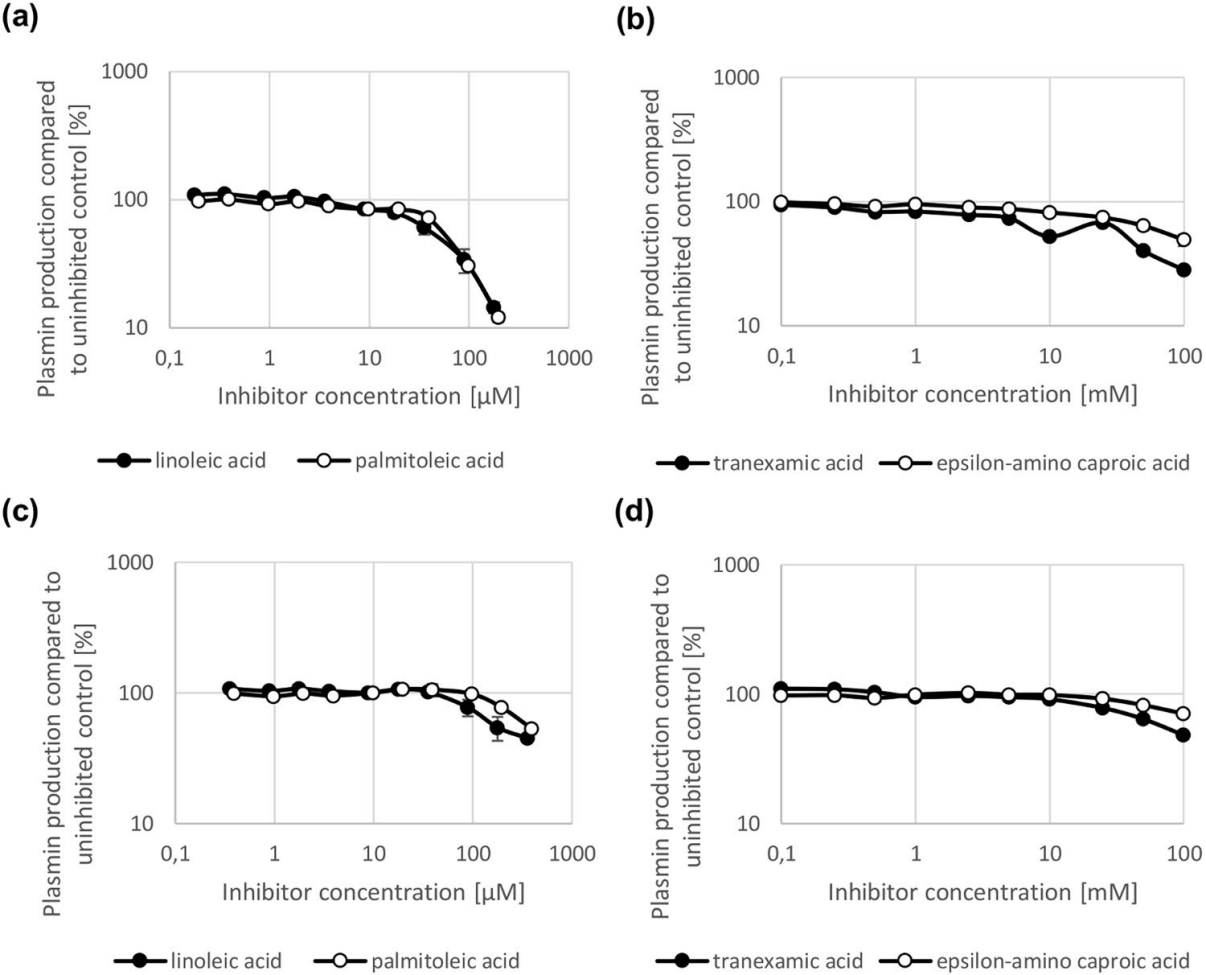
Inhibition of plasminogen activation in a cell-based plasminogen activation assay mediated by streptokinase cluster type 2a and 2b. LA and PA inhibit plasminogen activation mediated by streptokinase cluster type 2b (**a**) and 2a (**c**) in a concentration-dependent manner (micromolar range) in a cell-based plasminogen activation assay. By contrast, TXA and EACA only inhibit plasminogen activation by cluster type 2b (**b**) and 2a (**d**) at high concentrations (millimolar range). N = 3 for all experiments.

**Figure 3:**
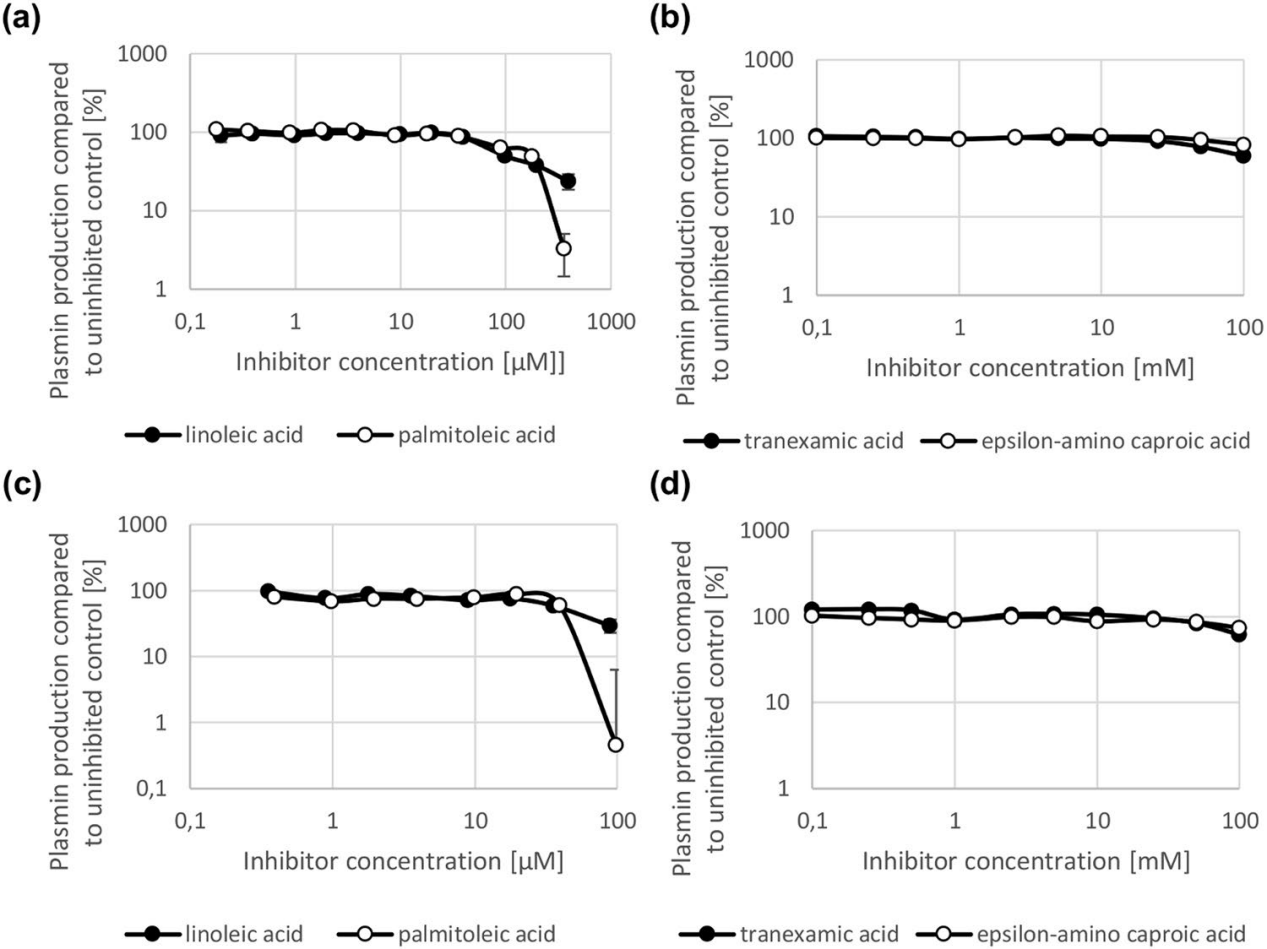
Inhibition of plasminogen activation in a supernatant-based plasminogen activation assay mediated by streptokinase cluster type 1 and 2a. LA and PA inhibit plasminogen activation mediated by streptokinase cluster type 2a (**a**) and 1 (**c**) in a concentration-dependent manner (micromolar range) in a supernatant-based plasminogen activation assay (**a**,**c**). By contrast, TXA and EACA only inhibit plasminogen activation mediated by streptokinase cluster type 2a (**b**) and 1 (**d**) at high concentrations (millimolar range) (**b**,**d**). N = 3 for all experiments.

**Figure 4:**
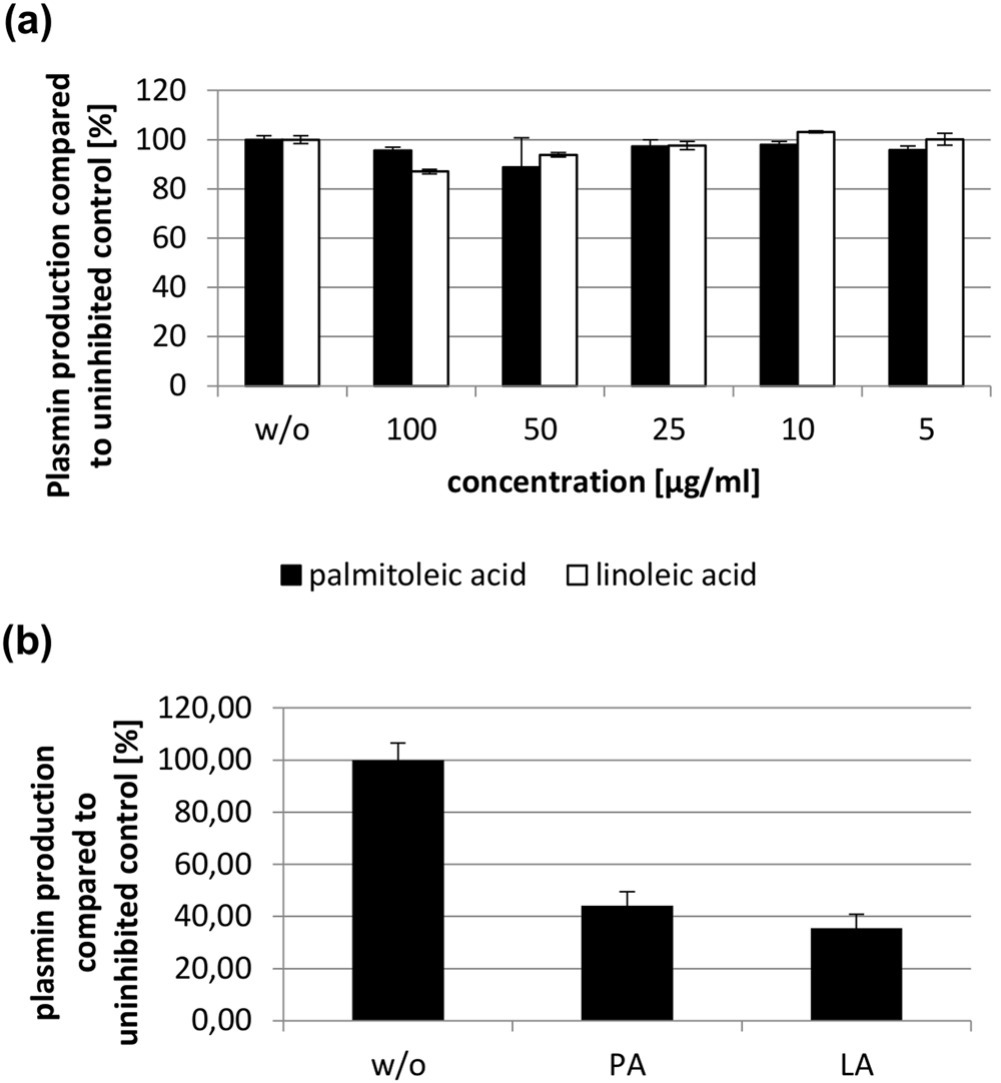
LA and PA do not inhibit staphylokinase-mediated plasminogen activation, but inhibit plasminogen activation by recombinant streptokinase. (**a**) Plasminogen activation using staphylokinase. Compared to the untreated (w/o) control neither palmitoleic nor linoleic acid show an effect. (**b**) Plasminogen activation using recombinant cluster 2a type streptokinase. Both palmitoleic (PA) and linoleic acid (LA) (concentration for both: 40 µg/ml) inhibit plasminogen activation mediated by recombinant cluster 2a type streptokinase. w/o: untreated control. N = 3 for all experiments.

With these two compounds, LA and PA, in hand the inhibition of different cluster types of streptokinase was assessed. Two clinically used drugs, ε-amino caproic (EACA) and tranexamic acid (TXA), interact with the kringle domains of plasminogen and thereby, inhibit its action^22–24^. Therefore, TXA and EACA (Fig. 1c,d) were used as controls to assess the inhibitory potential of LA and PA against streptokinase-mediated plasminogen activation. First, LA and PA were tested in cell-based plasminogen assays using cluster 2a and 2b type streptokinase. PA and LA both showed a 1000fold lower IC_50_ in a cell-based plasminogen activation assay using streptokinase cluster type 2b and a 250fold lower IC_50_ using cluster type 2a compared to TXA and EACA (Fig. 2, Table 1). Additionally, PA and LA were tested in supernatant-based plasminogen activation assays using cluster 1 and cluster 2a streptokinase. These assays rely upon secreted streptokinase activating plasminogen to plasmin^13^. In both assays (cluster 1 and cluster 2a type streptokinase), LA and PA had 500–1000 fold lower IC_50_-values than TXA and EACA, which only inhibited plasminogen activation marginally (Fig. 3, Table 1). Plasminogen activation was not inhibited by LA and PA upon activation with other activators of plasminogen such as staphylokinase (Fig. 4a). Therefore, LA and PA specifically inhibit *streptokinase-mediated* plasminogen activation. To exclude that the effects of streptokinase-mediated plasminogen activation were only attributable to killing of GAS upon treatment, we used recombinant cluster 2a type streptokinase. LA and PA at a concentration of 40 µg/ml were both able to achieve an inhibition of plasminogen activation of about 60–70% (Fig. 4b). Consequently, the effects of both fatty acids are attributable to the interaction with streptokinase and plasminogen. LA and PA inhibit the gain of plasmin activity by all cluster types of streptokinase so that GAS cannot disseminate so easily anymore.

**Table 1:** PA and LA show lower IC_50_-values than TXA and EACA in cell-based and supernatant-based plasminogen activation assays.

	PA	LA	TXA	EACA
**Cell-based**				
cluster 2b	61.1 µM (16.1 µg/ml)	64.5 µM (17.3 µg/ml)	100 mM	100 mM
cluster 2a	359.4 µM (119.8 µg/ml)	246.4 µM (82.1 µg/ml)	100 mM	>100 mM
**Supernatant-based**				
cluster 2a	91.6 µM (26.6 µg/ml)	156.2 µM (41.7 µg/ml)	>100 mM	>100 mM
cluster 1	40.9 µM (10.4 µg/ml)	46.6 µM (12.9 µg/ml)	>100 mM	>100 mM

IC_50_-values of PA, LA, TXA and EACA are displayed resulting from cell-based plasminogen activation assays with cluster 2a and cluster 2b type streptokinase and from supernatant-based plasminogen activation assays with cluster 2a and cluster 1 type streptokinase.

### Inhibition of streptokinase-mediated plasminogen activation is a unique feature of linoleic and palmitoleic acid

One would expect that other fatty acids may exhibit the same inhibitory potential as linoleic and palmitoleic acid. Therefore, seven commercially available fatty acids were tested in a cell-based plasminogen activation assay using cluster 2b and 2a type streptokinase and in a supernatant-based plasminogen activation assay using cluster 2a and cluster 1 type streptokinase. Among these seven fatty acids were four saturated (palmitic acid, stearic acid, myristic acid and arachidic acid) and three unsaturated (oleic acid, linolenic acid and arachidonic acid) fatty acids. Palmitic acid is a saturated C_16_ fatty acid and structurally close to PA whereas stearic acid is a saturated C_18_ fatty acid and structurally similar to LA. Myristic and arachidic acid are two saturated fatty acids which bear two C-atoms less and more, respectively, than PA and LA. Arachidonic acid is a polyunsaturated C_20_ fatty acid with double bonds at positions 5, 8, 11 and 14. Oleic acid, a C_18_ fatty acid with a double bond at position 9, and linolenic acid, a C_18_ fatty acid with three double bonds at position 9, 12 and 15, are both structurally related to LA and PA. All these fatty acids were tested in concentrations ranging up to 100 µg/ml (around 320–437 µM). Stearic, palmitic and arachidic acid showed unspecific marginal inhibitory effects which were not concentration-dependent on plasminogen activation via cluster 2b type streptokinase, whereas myristic acid, a C_14_ saturated fatty acid, as well as arachidonic, linolenic and oleic acid showed no effect (Fig. S7a). Regarding plasminogen activation via cluster 2a type streptokinase in a cell-based assay, no tested commercially available fatty acid showed an effect (Fig. S7b). Only oleic and linolenic acid showed marginal concentration-dependent inhibitory effects in a supernatant-based plasminogen activation assay via cluster 2a type streptokinase: linolenic acid inhibited plasminogen activation by 30% at 359 µM whereas oleic acid achieved around 45% inhibition at 354 µM (Fig. S7c). However, LA and PA achieved the same range of inhibition at 3–4 fold lower concentrations. Apart from arachidic, arachidonic and myristic acid which showed no effect, the other fatty acids showed an enhancement of plasminogen activation in a supernatant-based assay via cluster 1 type streptokinase (Fig. S7d). It can be concluded that the inhibitory activity of LA and PA regarding all cluster types of streptokinase-mediated plasminogen activation is not a common feature of unsaturated or saturated fatty acids in general, but unique to LA and PA.

### Linoleic and palmitoleic acid prolong entrapment of GAS in fibrin clots

**Figure 5:**
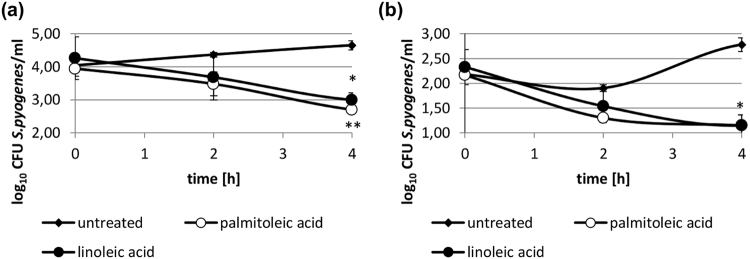
Prolongation of entrapment of streptococci within a clot upon treatment with LA and PA. Significantly fewer streptococci escape from a clot following treatment with LA or PA. (**a**) Streptococci harboring a cluster 2b type streptokinase were entrapped within a clot and treated with 60 µg/ml of either LA or PA or were left untreated. (**b**) Streptococci harboring a cluster 2a type streptokinase were entrapped within a clot and treated with 30 µg/ml of either LA or PA or were left untreated. cfu/ml in the supernatant were enumerated at 2 h and 4 h after treatment. *p < 0.05; **p < 0.01. N = 2 for all experiments.

When GAS infect tissue, they may be entrapped within a fibrin clot which can be dissolved through the streptokinase activation of plasminogen, paving the way for GAS to enter deeper tissue^25^. Hence, we investigated whether LA and PA prevented clot destruction by GAS producing either a cluster 2a or 2b type streptokinase. After 4 h, a significantly lower amount of GAS escaped from the clot treated with either LA and PA compared to the untreated clot (p < 0.05). This was observed in GAS strains harboring either cluster 2a or cluster 2b type streptokinase (Fig. 5).

### Linoleic and palmitoleic acid substantially prolong survival in a murine model

**Figure 6:**
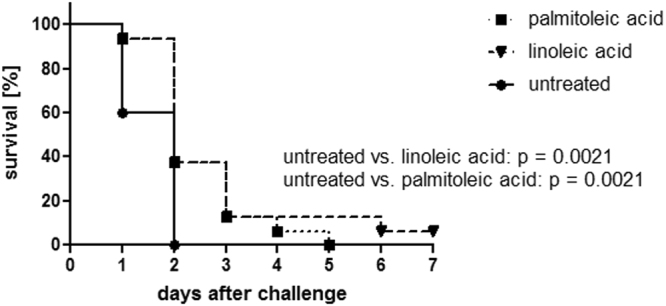
Significant prolongation of survival in an invasive murine infection model using *S. pyogenes* strain AP1. LA and PA significantly prolong survival in an invasive murine infection model using *S. pyogenes* strain AP1 (p < 0.01). Transgenic humanized plasminogen mice were infected with 1 × 10^7^ cfu AP1 s.c., treated with 50 µg of either PA (n = 16) or LA (n = 16) or PBS s.c. (n = 15) (the experiment was performed four times with cohorts of 3–5 mice for each group).

To assess the effects of LA and PA not only on the mechanistic and the *in vitro*-level, an invasive humanized plasminogen murine infection model was used. Mice were either administered PBS (untreated) or treated with LA or PA on the day of infection with *S. pyogenes* strain AP1^26^. Treated mice received a dose of 50 µg subcutaneously (approx. 2 mg/kg, average weight of mice 23 g) of LA or PA, respectively. Treatment with LA and PA significantly extended the survival time of the treated mice in comparison with the untreated group (p = 0.0021 for both treatment groups; Hazard ratio (Mantel-Haenszel) LA/untreated: 0.1429 and PA/untreated: 0.1429) (Fig. 6).

## Discussion

The deployment of plasmin as a GAS virulence factor during invasive disease is an established hypothesis^6,7^. In this study, we present two fatty acids, LA and PA, displaying inhibitory activity against streptokinase-mediated plasminogen activation and extending survival in a lethal murine model of GAS invasive infection.

Plasminogen can be bound directly to GAS surface receptors such as SEN, GAPDH, PAM and Prp^27–30^. Free or surface-bound plasminogen can then be activated to plasmin through the action of streptokinase. Acquisition of surface plasmin activity facilitates dissemination into deeper tissue^31,32^. Several myxobacterial extracts tested in this study inhibited activation to plasmin through the action of streptokinase. Using NMR, HPLC-fractionation and GC-MS we identified these inhibiting compounds as LA and PA.

We have shown inhibition of plasminogen activation mediated by all cluster types of streptokinase is unique to LA and PA. Using saturated fatty acids with the same chain length as LA and PA, such as palmitic or stearic acid, had no effect regarding inhibition of plasminogen activation. Addition or reduction of the chain length, in the case of myristic or arachidic acid, did not result in inhibition activity, either. Arachidonic acid, as a polyunsaturated C_20_ fatty acid, was again not active. By contrast, oleic acid, a C_18_ fatty acid with a double bond at position 9, showed slight inhibitory activity in the case of supernatant-based plasminogen activation via cluster 2a type streptokinase. However, oleic acid was not active in a cell-based plasminogen activation assay with cluster 2a type streptokinase and might, therefore, not be a good choice for an *in vivo* therapeutic. Linolenic acid, bearing an additional double bond at position 15 compared to LA, showed similar effects as oleic acid, although these were weaker. Consequently, one or two double bonds are necessary for activity – saturation of fatty acids leads to complete loss of activity, even if chain length is augmented or reduced (as seen for myristic, palmitic, stearic and arachidic acid). Three double bonds, as in the case of linolenic acid, resulted in reduction of activity compared to LA. Maintaining the chain length of LA, but saturating one double bond, as in case of oleic acid, results in weak activity in only one assay against one cluster type. More than three double bonds, as in the case of arachidonic acid, resulted in complete loss of activity. Regarding the structure-activity-relationship of the fatty acids, two double bonds (in position 9 and 12) for the C_18_ fatty acid are essential to prevent loss of activity. Moreover, saturation of fatty acids and variation of the chain length do not result in enhanced activity. For the C_16_ fatty acid, a double bond in position 9 is necessary to result in the same activity as detected for LA. However, one might speculate whether another location of the double bond in a C_16_ fatty acid could have the same effect as seen for PA. This has not been tested in this study as these fatty acids were not easily commercially available.

Plasminogen activation mediated by staphylokinase was not inhibited, suggesting that the inhibitory capacity of LA and PA is specific to streptokinase. Notably, the activity of GAS surface-bound plasmin or plasminogen was inhibited by LA and PA. Compared to EACA and TXA, the activity of LA and PA with respect to inhibition of different cluster types of streptokinase was 500–1000 fold higher. Clot lysis relies on the activation of plasminogen to plasmin. Both LA and PA inhibit plasmin activity directly. GAS escape from clots by activating plasminogen following the secretion of streptokinase and/or by binding plasmin to their cell surface^25,33,34^. By inhibition of streptokinase-mediated plasminogen activation and thereby inhibiting the gain of plasmin activity, GAS escape from clots is hampered as clot lysis relies on plasmin activity.

GAS escape from clots by activating plasminogen following secretion of streptokinase and/or capturing plasmin at the bacterial cell surface^25,33,34^. This mechanism is also active *in vivo* ^35^: Sun and colleagues showed that streptokinase-deficient strains were not able to cause death in human plasminogen transgenic mice. In addition, they showed that lack of fibrinogen, essential for clot formation and entrapment of GAS *in vivo*, resulted in a faster dissemination upon subcutaneous infection with GAS^6^. Therefore, it is likely that inhibition of streptokinase might hamper dissemination of GAS into deeper tissue and aid in immune clearance^9^. However, we can only speculate that the bacterial loads of GAS upon treatment are lower on day 1 or 2 after challenge compared to the control group as our *in vitro* results demonstrate that GAS were entrapped in clots for a longer time and, therefore, as observed by Sun *et al*. were hindered to disseminate^6^. Mice survived longer upon treatment with LA or PA. Thus, we demonstrate here that targeting streptokinase-mediated plasminogen activation during GAS invasive infection has therapeutic potential^36^ – similar to the targeting of streptokinase gene expression^18,19,37^. Sun and colleagues found two inhibitors (targeting *inter alia* streptokinase gene expression) with an IC_50_ of around 100 µM. One of these inhibitors showed significant effects in an *in vivo* model with a survival rate of around 40%^18^. Compared to these inhibitors, LA and PA showed quite low IC_50_-values and a significant effect on survival in a lethal murine infection model – although LA and PA were only administered once whereas the compounds of Sun and colleagues were administered four to five times daily^18^. Current standard of care treatment options still result in mortality rates of around 20–30% and a timely diagnosis of a soft tissue infection is still the key to prevent death^38,39^. Therefore, it will be interesting to explore the possible effect of co-administration of LA and PA or of using LA or PA in conjunction with the standard of care treatment^38^ in future studies.

In summary, we present linoleic and palmitoleic acid isolated from myxobacteria as fatty acids effectively inhibiting *streptokinase*-mediated plasminogen activation of all cluster types. This feature is not inherited by other, structurally similar fatty acids, e.g. oleic or linolenic acid. LA and PA prevent clot destruction and escape by GAS and significantly extend survival in a lethal invasive disease model. Consequently, LA and PA can be considered as a starting point for an anti-virulence strategy to combat the threat of GAS invasive infections.

## Methods

### Bacterial strains, media and growth conditions

The group A streptococcal strains AP1 (clinical isolate, originally: 40/58 strain from the WHO Collaborating Center for references and research on Streptococci, Institute of Hygiene and Epidemiology, Prague, Czech Republic, cluster 2a type streptokinase, GenBank accession no. CP007537.1^40^), A614 (clinical isolate from an invasive infection, cluster 2a type streptokinase, GenBank accession no. KT895340), A666 (clinical isolate from a patient suffering from toxic streptococcal shock syndrome, cluster 2b type streptokinase, GenBank accession no. KT895341), A314 (NS1133, cluster 2b type streptokinase^13^) and A328 (NS836, cluster 1 type streptokinase^13^) were obtained from the strain collection of the department of Medical Microbiology (MMIK) within the Helmholtz Centre for Infection Research (HZI). Streptococcal strains were inoculated onto sheep blood agar plates and were incubated overnight at 37 °C. The following day the THY medium was inoculated with the respective streptococcal strain and incubated overnight at 37 °C.

### Compounds, reagents, myxobacterial extracts and compounds

TXA and EACA were purchased from Sigma. LA and PA were isolated from myxobacteria. In addition, LA and PA were purchased from Acros Organics. Oleic, arachidic, myristic, palmitic, stearic, linolenic acid were purchased from Acros Organics. Arachidonic acid was purchased from Sigma. Fatty acids were treated as follows to assure that they are protonated: every fatty acid was dried under nitrogen flow at 40 °C. Then they were dissolved in methanol containing 5% H_2_O and 10% HCOOH. The methanol phase was extracted 5 times with n-heptane. The n-heptane phases were separated from the methanol phase, united and dried under nitrogen flow at 40 °C. Fatty acids as well as TXA and EACA were dissolved and diluted in PBS for biological testing. Myxobacterial extracts and compounds for screening purposes were obtained from the Departments of Microbial Natural Products (MINS) and of Microbial Drugs (MWIS) within the Helmholtz Centre for Infection Research (HZI).

### Analytical methods

Myxobacterial extracts were analyzed by RP-HPLC on an Agilent 1260 system (Agilent Technologies) equipped with a diode array UV detector and a Corona ultra-detector (Dionex). 1 µl of each extract was injected. The following conditions were used: column Nucleodur 120 EC, 125 × 2 mm, 5 µm, C_18_ (Macherey-Nagel); temperature: 40 °C; solvent A: 95% H_2_O, 5% acetonitrile, 5 mM ammonium acetate, 40 µl/l acetic acid; solvent B: 95% acetonitrile, 5% H_2_O; 5 mM ammonium acetate, 40 µl/l acetic acid; gradient 10–100% B for 30 min, 100% B for 10 min; 10% B post-run for 10 min; flow 0.3 ml/min; UV detection 200–450 nm. Fractions obtained after fermentation and during purification of fermenter products were analyzed by analytical RP-UPLC on an Agilent 1260 HPLC system (Agilent Technologies) equipped with a diode array UV detector and a corona ultra-detector (Dionex). 1 µl of each fraction was injected. The following conditions were used: column Waters Acquity UPLC BEH C18, 130 Å, 1.7 µm, 50 × 2.1 mm; temperature: 40 °C; solvent A: 95% H_2_O, 5% acetonitrile, 5 mM ammonium acetate, 40 µl/l acetic acid; solvent B: 95% acetonitrile, 5% H_2_O; 5 mM ammonium acetate, 40 µl/l acetic acid; gradient: 90% A for 10 min, 10–100% B for 30 min, 100% B for 10 min; 10% B post-run for 10 min; flow 0.3 ml/min; UV detection 200–450 nm. In addition, linoleic and palmitoleic acid were analyzed by analytical RP-UPLC under the same conditions. Fractionation via HPLC was performed analogously to RP-HPLC. For fractionation, the Corona ultra-detector (Dionex) was replaced by an Agilent fraction-collector. Fractions were collected every 30 sec in a 96 well-plate. At the end of the HPLC run the plate containing the liquid fractions was dried under nitrogen flow at 37 °C for 1 h. For fractionation of RC-fractions the following conditions were used: column Waters Acquity UPLC BEH C18, 130 Å, 1.7 µm, 50 × 2.1 mm; temperature: 40 °C; solvent A: 95% H_2_O, 5% acetonitrile, 5 mM ammonium acetate, 40 µl/l acetic acid; solvent B: 95% acetonitrile, 5% H_2_O; 5 mM ammonium acetate, 40 µl/l acetic acid; gradient 10–100% B for 30 min, 100% B for 10 min; 10% B post-run for 10 min; flow 0.3 ml/min; UV detection 200–450 nm.

### Fermentation of the myxobacterial strain 706

The strain 706^20^ was cultivated in 100 ml of Cy/H medium (Cy medium: 0.3% casitone, 0.1% yeast extract, 0.1% CaCl_2_, 50 mM HEPES, 1.6% agar, pH 7.2; H medium: 0.2% soya flour, 0.2% glucose, 0.8% starch, 0.2% yeast extract, 0.1% CaCl_2_, 0.1% MgSO_4_, 50 mM HEPES, 8 mg/L Fe-EDTA, pH 7.4; Cy/H medium is a mixture from Cy and H 1:1) in a 250 ml flask at 30 °C, 180 rpm and amplitude 50 mm. For production of metabolites, it was inoculated 1:10 in 1 l of Pol medium (0.3% soluble starch, 0.3% probion (single cell protein, Hoechst AG), 0.05% CaCl_2_, 0.2% MgSO_4_, 50 mM HEPES, pH 7.2) in ten 2.5 l flasks at 30 °C, 180 rpm and amplitude 50 mm. The strain 706 was cultivated in a fermenter in 70 l of Pol medium supplemented with 0,1% Amberlite XAD-16 adsorber resin (Sigma) at 30 °C. The pH was regulated to 7.2 ± 0.1 with titration by KOH (2.5% (w/w)) and H_2_SO_4_ (2.5% (w/w)). The dissolved oxygen was kept above 20% of saturation by increasing the stirrer (rushton turbine) speed and an aeration rate of 0,03 vvm. The minimum of rotation was 100 rpm. The fermenter was inoculated with 8 l of culture of the strain 706. Every day samples of 100 ml were taken for analysis of activity. The fermenter was harvested on day 9.

### Purification of fatty acids after fermentation

588 g of Amberlite XAD-16 adsorber resin (Sigma) and some residual cells were extracted in a column with 2.5 l acetone. The acetone was evaporated using a rotavapor (Heidolph). The residue was dissolved in H_2_O and extracted three times with ethyl acetate. The ethyl acetate fraction (RC 22; 5.2 g) was evaporated. Before proceeding with further purification RC 22 and the H_2_O were tested for biological activity in a facilitated plasminogen activation assay. Then RC 22 was dissolved in methanol containing 5% H_2_O and extracted three times with n-heptane. The methanol-phase was termed RC 23. Fraction RC 23 (2.6 g) was evaporated and dissolved in methanol. The extracted n-heptane fraction (RC 24; 2.4 g) was evaporated as well. For testing biological activity RC 24 was dissolved in methanol. Afterwards, RC 23 and RC 24 were tested for biological activity in the facilitated plasminogen activation assay. RC 23 (dissolved in methanol containing 5% H_2_O) was separated by Sephadex LH-20 chromatography (column 7 × 60 cm; flow 5.8 ml/min; UV detection 254 nm, solvent: 95% methanol, 5% H_2_O). Fractions (RC 23.x, with x = fraction number) were collected for peaks, shoulders, valleys and baseline of the chromatogram and were tested for biological activity in the facilitated plasminogen activation assay. RC 24 (dissolved in dichloromethane) was separated by Si-Flash chromatography using a Grace Reveleris system (column Reveleris Silica 40 g; solvent A dichloromethane; solvent B acetone; gradient: 4% B A for 1 min, 4–10% B for 20 min, 10% B for 10 min, 10–100% B for 10 min, 100% B for 25 min; flow 15 ml/min; UV detection 210 nm, 254 nm, 280 nm; ELSD detection (ELSD threshold 5 mV, sensitivity low)). Fractions (RC 24.x, with x = fraction number) were collected for peaks, shoulders, valleys and baseline. Fractions were evaporated, dissolved in methanol and then tested for biological activity in the facilitated plasminogen activation assay. 1 g of the fraction RC 23.8 (as it was active in the facilitated plasminogen activation assay) dissolved in methanol was separated by RP-MPLC (column 480 × 30 mm; Nucleodur C_18_ [Macherey-Nagel]; solvent A: 50% H_2_O/50% MeOH + 5% HCOOH; solvent B: 100% MeOH + 5% HCOOH; gradient: 75% B for 50 min, 75–100% B for 5 min; 100% B for 30 min; flow 30 ml/min; UV detection 205 nm). Fractions (RC 25.x, with x = fraction number) were collected for peaks, shoulders and valleys of the chromatogram. Fractions were evaporated, dissolved in methanol and then tested for biological activity in the facilitated plasminogen activation assay. For further purification RC 25.1 and RC 25.3 (as both fractions were active in the facilitated plasminogen activation assay) were separated by Si-Flash chromatography using a Grace Reveleris system (column Reveleris Silica HP 4 g (20 µm); solvent A dichloromethane; solvent B acetone; gradient: 100% A for 4 min, 0–5% B for 10 min, 5% B for 5 min, 5–100% B for 10 min, 100% B for 25 min; flow 15 ml/min; UV detection 210 nm, 220 nm, 254 nm; ELSD detection (ELSD threshold 5 mV, sensitivity low)). Fractions were collected for peaks, shoulders, valleys and baseline. Fractions were evaporated, dissolved in methanol and then tested for biological activity in the plasminogen activation assay. Fractions obtained from RC 25.1 were termed RC 26.x (with x = fraction number) and those from RC 25.3 RC 27.x (with x = fraction number). Active fractions of RC 26 and RC 27 were united in correlation with the data from the facilitated plasminogen activation assay and termed RC 28.1/RC 36.1 and RC 28.5/RC 36.2.

### Structure elucidation via NMR and HR-ESIMS

^1^H and ^13^C NMR spectra of RC 36.1 and RC 36.2, linoleic acid, palmitoleic acid, linolelaidic acid, palmitelaidic acid in CDCl_3_ were recorded on a Bruker Avance 700 MHz spectrometer, locked to the deuterium signal of the solvent. Chemical shifts are given in parts per million (ppm) and coupling constants in Hz. HR-ESIMS data of RC 36.1 and RC 36.2 were recorded on a Maxis ESI TOF mass spectrometer (Bruker Daltonics). Molecular formulas were calculated including the isotopic pattern (Smart Formula algorithm). HR-ESIMS data of RC 36.1 (palmitoleic acid) (C_16_H_30_O_2_): *m/z* 255.2327 [M + H]^+^ (calcd. 255.2318); *m/z* 237.2217 [M-H_2_O + H]^+^ (calcd. 237.2213); *m/z* 509.4575 [2 M + H]^+^ (calcd. 509.4564); *m/z* 491.4468 [2M-H_2_O + H]^+^ (calcd. 491.4458). HR-ESIMS data of RC 36.2 (linoleic acid) (C_18_H_32_O_2_): *m/z* 281.2477 [M + H]^+^ (calcd. 281.2475); *m/z* 263.2372 [M-H_2_O + H]^+^ (calcd. 263.2369); *m/z* 561.4884 [2 M + H]^+^ (calcd. 561.4877); *m/z* 543.4773 [2M-H_2_O + H]^+^ (calcd. 543.4772).

### Methods for location of double bond(s) via GC-MS

GC-MS spectra were recorded on a Trace GC Ultra (Thermo Scientific) equipped with a split/splitless injector coupled to an ITQ 900 (Thermo Scientific; Ion Trap). One µl of each sample was injected in split mode using a PAL Combi-XT autosampler. Base temperature of the injector was 275 °C with a split flow of 10 ml/min. Helium was used as carrier gas with a flow of 1 ml/min. A TraceGOLD^TM^ TG-5MS column was used (30 m × 0.25 mm × 0.25 µm, Thermo Scientific). Mass spectrometric conditions were as follows: The source temperature was held at 200 °C during GC-MS runs. The mass selective detector was operated in full scan mode with positive polarity; spectra were acquired in the range of *m/z* 40–700; maximal ion time was 25 ms. Scan control, data acquisition and processing were performed with XCalibur Software (Thermo Scientific). Fatty acids (RC 36.1, RC 36.2) were treated with 20 µl thionyl chloride for 10 min at 100 °C to generate the acid chlorides and dried under nitrogen flow at 40 °C. Under cooling to 0 °C 500 µl of a freshly prepared solution of 2-amino-2-methyl-propan-1-ol (Sigma) in dichloromethane (10 mg/ml) were added to each sample. Then samples were left on ice for 1 h. Afterwards, they were kept at room temperature for 1 h before they were dried under nitrogen flow at 40 °C. 500 µl of trifluoro acetic anhydride (Sigma) were added to each sample. After 3 h at room temperature, they were dried under nitrogen flow at 40 °C. Samples were dissolved in 2.14 ml iso-hexane and 0.86 ml water. The organic layer was transferred into a new vial. The water phase of each sample was extracted two more times with iso-hexane. All organic phases were united in a new vial and the solvent was evaporated under nitrogen flow at 40 °C. Samples were dissolved in 100 µl of iso-hexane and analyzed by GC-MS using the following gradient: 80 °C as initial temperature for 3 min, 80–180 °C ramped with 20 degrees/min, 180–280 °C ramped with 2 degrees/min, 280 °C held for 15 min, 280–325 °C ramped with 5 degrees/min, 325 °C held for 5 min.

### Facilitated plasminogen activation assay for testing of myxobacterial extracts and compounds

Supernatant of an overnight culture of the GAS strain A614 was used as this strain secretes a cluster 2a type streptokinase. 75 µl of the culture supernatant was added to the wells of a 96well plate followed by 69 µl PBS, 5 µl S-2251 (10 mM stock solution, chromogenic substrate for the detection of plasmin, Chromogenix) and 1 µl plasminogen (1 mg/ml stock solution, Biopur or Sigma). 1 µl of each myxobacterial extract or each myxobacterial compound (dissolved in methanol, 1 mM) was added to test possible inhibition of plasminogen activation. The assay was run at 37 °C. The absorption was measured at the beginning of the reaction and then every hour at 405 nm after 3 sec of shaking on a Tecan Sunrise ELISA Reader using Magellan software. The reaction was finished when untreated samples had reached an OD_405nm_ of about 1.

### Plasminogen assay with staphylokinase as activator of plasminogen

The assay was conducted as described above using staphylokinase (Cedarlane Labs) as an activator of plasminogen (staphylokinase final concentration 2 µg/ml per well in 75 µl of PBS. Stocks of LA and PA were prepared in methanol and then diluted in PBS. The untreated control received the same amount of methanol diluted in PBS as in case of treatment with PA or LA. Each experiment was repeated three times.

### Cell-based plasminogen activation assay

The pellet from a mid-log phase culture of the strain A614 (cluster 2a type) or A314 (cluster 2b type) was washed twice with PBS and bacteria were adjusted to an OD_600_ of 1.0 in HEPES-buffer (10 mM HEPES, 150 mM NaCl, 0.01% Tween 20, pH 7.4). 90 µl of bacterial cells, plasminogen (final concentration 6.7 µg/ml per well) and fibrinogen (final concentration 1 µM per well) were pre-mixed. Then inhibitory compounds (LA, PA, oleic, myristic, stearic, palmitic, arachidonic, arachidic, linolenic acid, TXA or EACA) were added. Stocks of LA and PA were prepared in methanol and then diluted in PBS. Stocks of arachidic, oleic, arachidonic, myristic, palmitic, stearic and linolenic acid were prepared in 60% DMSO/PBS and then diluted in PBS. The untreated control received the same amount of PBS as in case of TXA and EACA as control or the same amount of methanol diluted in PBS as in case of LA and PA, respectively, or the same amount of DMSO diluted in PBS as in case of the other commercially available fatty acids. Then 50 µl of the respective supernatant (supernatant from the strain A614 if cells from A614 were used, supernatant from the strain A314 were used in case of cells from the GAS strain A314) and 3.75 µl 10 mM S-2251 (Chromogenix) were added. The assay was incubated at 37 °C and measured at the beginning of the reaction and then every hour at 405 nm after 3 sec of shaking on a Tecan Sunrise ELISAReader using Magellan software. Each experiment was repeated three times. IC_50_ values were calculated using Microsoft® Excel software.

### Supernatant-based plasminogen activation assay

An overnight culture of the strain A328 (cluster 1 type) or A614 (cluster 2a type) was diluted 1:10 in pre-warmed THY medium and grown to an OD_600_ of around 0.5 at 37 °C. Bacteria were centrifuged at 2123 × *g* for 10 min and the supernatant was separated from the pellet. The pellet was resuspended in Tris-HCl buffer (50 mM Tris pH 7.5). Then inhibitory compounds (LA, PA, oleic, myristic, stearic, palmitic, arachidonic, arachidic, linolenic acid, TXA or EACA) were added. Stocks of TXA and EACA were prepared in PBS. Stocks of LA and PA were prepared in methanol and then diluted in PBS. Stocks of arachidic, oleic, arachidonic, myristic, palmitic, stearic and linolenic acid were prepared in 60% DMSO/PBS and then diluted in PBS. The untreated control received the same amount of PBS as in case of TXA and EACA as control or the same amount of methanol diluted in PBS as in case of LA and PA, respectively, or the same amount of DMSO diluted in PBS as in case of the other commercially available fatty acids. 20 µl of the respective supernatant was added to fibrinogen (in case of cluster 2a type streptokinase, final concentration 1 µM per well), plasminogen and S-2251 in Tris-HCl buffer. The assay was run at 37 °C. The absorption was measured at the beginning of the reaction and then every hour at 405 nm after 3 sec of shaking on a Tecan Sunrise ELISAReader using Magellan software. Each experiment was repeated three times. IC_50_ values were calculated using Microsoft® Excel software.

### Cloning, overexpression and purification of recombinant streptokinase

An overnight culture of the strain A614 (in THY medium) was centrifuged at 5000 rpm for 10 min. The supernatant was discarded and the pellet was resuspended in TE-buffer (from DNeasy blood and tissue kit, Qiagen) containing 10% DNase-free RNase (10 µg/ml). The suspension was transferred to a vial filled with zirconium beads. Bacterial cells were lysed using a fastprep machine (MP Biomedical) twice for 30 sec at 4.0 m/sec. Then AL-buffer (from DNeasy blood and tissue kit, Qiagen) was added and beads were spun down for 30 sec. The lysate was transferred to a new reaction tube and proteinase K was added. The tube was inverted and incubated for 30 min at 56 °C. Then 100% ethanol was added and genomic DNA was isolated according to the protocol of the DNeasy blood and tissue kit (Qiagen). Then a PCR was performed to amplify the *ska* gene. Table S3 shows the sequences of the primers as well as the reaction conditions. Using a gel electrophoresis, the 1500 bp product was detected, cut out and purified using a PCR clean-up, gel extraction kit (Machery-Nagel). Afterwards, the PCR product was digested using the restriction enzymes BamH1 and SaL1 for 3 h at 37 °C. FastDigest® enzymes and reaction buffer (both from Fermentas) were used. Then an agarose gel of the digested product was run and the band was cut out and purified using a PCR-clean-up, gel extraction kit (Macherey-Nagel). The *E.coli* strain DH5α-pQe30-TEV (from the MMIK department’s strain collection, HZI Braunschweig^41^) was inoculated from a glycerol culture in LB-medium containing a final concentration of 100 µg/ml ampicillin and incubated at 180 rpm at 37 °C overnight. The following day the pQe30-TEV vector was isolated using a QiaPrep Spin MiniPrep Kit (Qiagen). Then the vector was digested using the same digestion conditions as described above. After digestion, an agarose gel electrophoresis of the product was performed; the vector was cut out and purified as described for the PCR product. The digested PCR product of the *ska* gene were ligated into the pQe30-TEV vector using T4-ligase. The reaction was run for 16 h at 17 °C. Ligation efficiency was checked by PCR. The vector including the *ska* gene was then transformed into *E. coli* strain DH5α using heat shock at 43 °C for 1 min. A clone containing the *ska* gene (confirmed by PCR) was then cultivated in LB medium containing 200 µg/ml ampicillin and expression of *ska* gene was induced for 4 h at 30 °C and 120 rpm using IPTG (1 mM) after the strain had reached an OD of 0.5–0.6. Then bacterial cells were centrifuged using a Sorvall centrifuge at 3000–8000 rpm at 4 °C and washed twice with cold PBS. Bacterial cells were then treated with lysis buffer and protease inhibitor before a FrenchPress was used to lyse bacterial cells. Then cell lysate was centrifuged at 13.000 rpm for 15 min at 4 °C. The supernatant was loaded onto a Ni-proteino column (Macherey-Nagel) and the protein was purified following the manufacturer’s protocol. Then the recombinant streptokinase was dialyzed in PBS overnight.

### Plasminogen assay using recombinant streptokinase

75 µl of the recombinant streptokinase (concentration 1 mg/ml) was added to the wells of a 96well plate followed by 69 µl PBS, 5 µl S-2251 (10 mM stock solution, chromogenic substrate for the detection of plasmin, Chromogenix) and 1 µl plasminogen (1 mg/ml stock solution, Biopur or Sigma). LA and PA (both dissolved in methanol) were added at a final concentration of 40 µg/ml per well. The untreated control was treated with the same amount of methanol as used for dissolving LA and PA. The assay was run at 37 °C. The absorption was measured at the beginning of the reaction and then every hour at 405 nm after 3 sec of shaking on a Tecan Sunrise ELISA Reader using Magellan software. The reaction was finished when untreated samples had reached an OD_405nm_ of about 1.

### Clotting assay

To assess whether LA and PA influence the entrapment of bacteria within clots, a 50 ml overnight culture (THY medium) of the *S. pyogenes* strains AP1 or A666 was centrifuged at 4332 × *g* for 10 min. The supernatant was discarded and the pellet was washed twice in 12.9 mM sodium citrate buffer. Then the pellet was dissolved in 1 ml of 12.9 mM sodium citrate buffer and the bacteria were adjusted to a transmission of 10% at 600 nm. The suspension was diluted 1:100 in 12.9 mM sodium citrate buffer. For clot induction, 100 µl citrate plasma, 100 µl of the respective bacterial suspension and 50 µl thrombin-reagent (Haemochrom Diagnostica) were used. When clots were formed, 1 ml of 1% plasma in 12.9 mM sodium citrate buffer was added to each sample. Then clots were left untreated (treated with sodium citrate buffer only) or were treated with LA or PA (diluted in sodium citrate buffer). For clots formed with *S. pyogenes* strain A666, a final concentration of 60 µg/ml was used for both, LA and PA. For those formed with *S. pyogenes* strain AP1, a final concentration of 30 µg/ml was used. 50 µl of the supernatant of each sample were plated onto blood agar plates in serial dilutions at 0, 2 and 4 h after clot induction and incubated at 37 °C overnight. Colonies were counted the following day to assess the number of bacteria which had escaped the clot. Data are displayed as log_10_ cfu of the respective strain per ml. Two independent experiments were performed. Standard deviations were calculated accordingly. Citrate plasma was obtained from healthy volunteers who gave their informed consent.

### Animal experiments

All animal experiments were performed in strict accordance with the guidelines of the “European Convention for the Protection of Vertebrate Animals used for Experimental and other Scientific purposes”, with the “Australian Code of Practice for the Care and Use of Animals for Scientific Purposes” and with the “Guidelines for the Care and Use of Laboratory Animals” (National Health and Medical Research Council, Australia). All experiments conducted in Germany were approved by the ethical board of “Niedersächsisches Landesamt für Verbraucherschutz und Lebensmittelsicherheit”, Oldenburg, Germany (animal ethics no.: 33.9-42502-04-12/1009). All experiments conducted in Australia were approved by the University of Queensland Animal Ethics Committee, Brisbane, Australia (animal ethics no.: SCMB/ 553/12/NHMRC). Humanized Plg transgenic *AlbPLG1* mice, heterozygous for the human Plg gene^6^, were bred and used in the animal facility at the Helmholtz Centre for Infection Research or at the Australian Infectious Disease Research Centre. An overnight culture of the strain AP1 (in THY medium) was re-inoculated in fresh THY medium and grown until mid-log phase (OD 0.4–0.6). Then the bacteria were centrifuged at 4332 × *g* for 10 min and washed twice in PBS. They were resuspended in PBS and adjusted to an OD of 1.0. Sex- and age-matched (eight- to sixteen-week-old) groups of C57Bl6/J huPlg positive transgenic mice were infected s.c. with 1 × 10^7^ cfu of *S. pyogenes* strain AP1. Cohorts of 15–16 mice were used (the experiment was performed four times with cohorts of 3–5 mice). The specific inoculum dose was administered in a 100 µl volume. Untreated mice received PBS subcutaneously (s.c.) as a control on the day of infection. Treated mice received either 50 µg of linoleic acid (diluted in PBS) or 50 µg of palmitoleic acid (diluted in PBS) s.c. on the day of infection. During the experiment mice were monitored for locomotion, behavior, appearance and weight. Mice were euthanized when they lost more than 20% of weight or when they reached a score of two for three of the following parameters: locomotion, appearance or behavior (authority regulations). The experiment ended after 7 days or after all mice had died.

### Statistical analysis

Significance was determined by using a two-tailed, paired student’s t-test. Survival data were analyzed using GraphPad Prism7 Software. Significance was determined by using a Mantel-Cox- and a Wilcoxon-Test.

## Electronic supplementary material

Supplementary Information
