# Supplementary material for: Linoleic and palmitoleic acid block streptokinase-mediated plasminogen activation and reduce severity of invasive group A streptococcal infection
Source: Sci Rep. 2017 Sep 18;7:11798. doi: 10.1038/s41598-017-11276-z (PMC5603603; doi:10.1038/s41598-017-11276-z)
Supplement: Supplementary file 1 — Supplementary Information [file 41598_2017_11276_MOESM1_ESM.pdf]

# Linoleic and palmitoleic acid block streptokinase-mediated plasminogen activation and reduce severity of invasive group A streptococcal infection

Katharina Rox[1,2,3,6], Rolf Jansen[4,6], Torsten G. Loof[2,5], Christine M. Gillen[7], Steffen Bernecker[4,6], Mark J. Walker[7], Gursharan Singh Chhatwal [2†], Rolf Müller[1,6*]

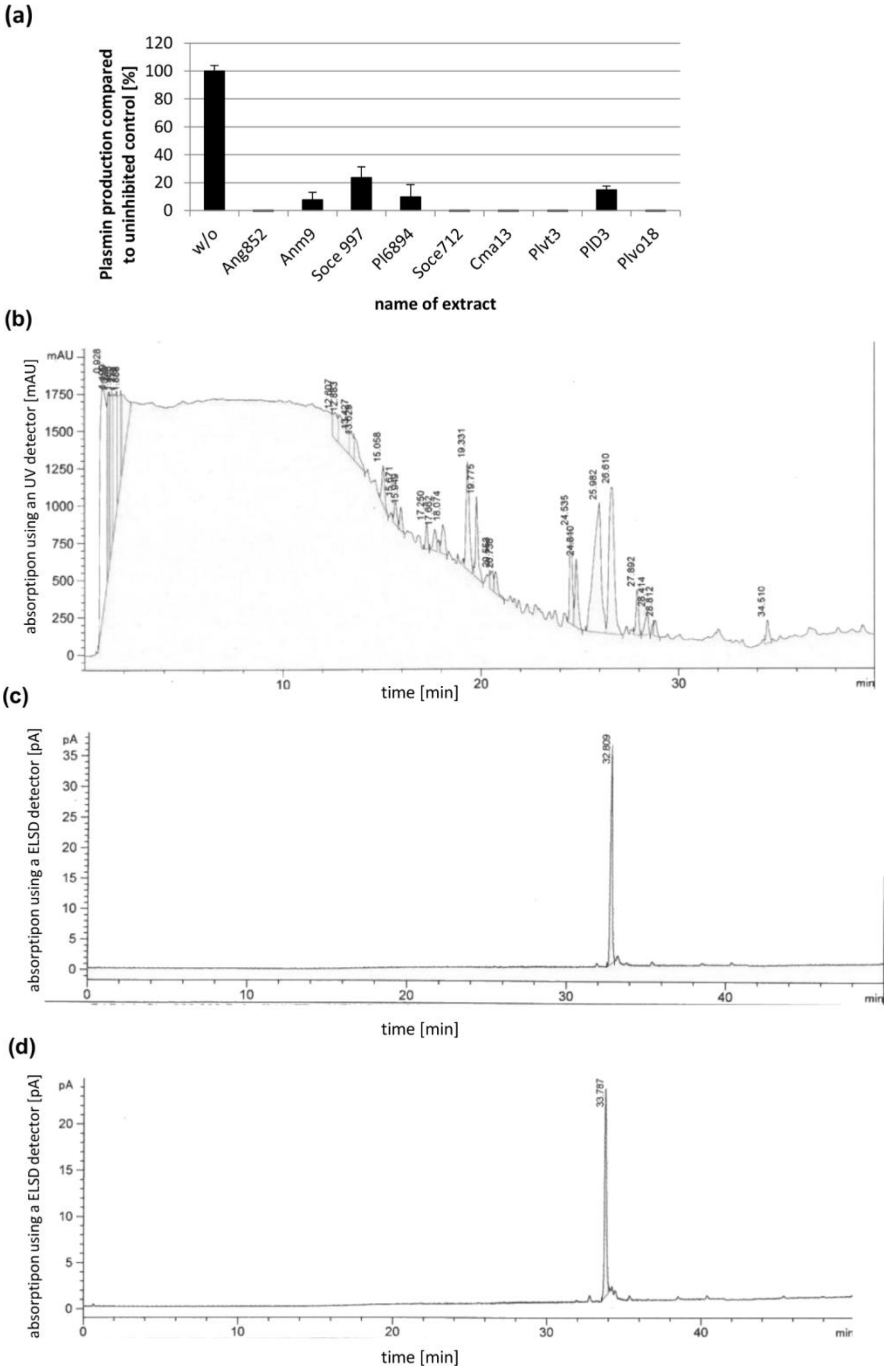


**Figure S1.** (a) Several myxobacterial extracts inhibit plasminogen activation mediated by a cluster 2a type streptokinase. Extract names are displayed according to the code of our internal database. Plasmin production in the absence of an inhibitor is designated by w/o. Plasmin production in the presence of inhibiting extracts is expressed as a percentage of the uninhibited control. (b) Representative chromatogram of the HPLC-fractionation of one inhibitory myxobacterial extract (Anm9). (c,d) HPLC-chromatograms of RC 36.1(c) and RC 36.2 (d) after isolation by activity-guidance.

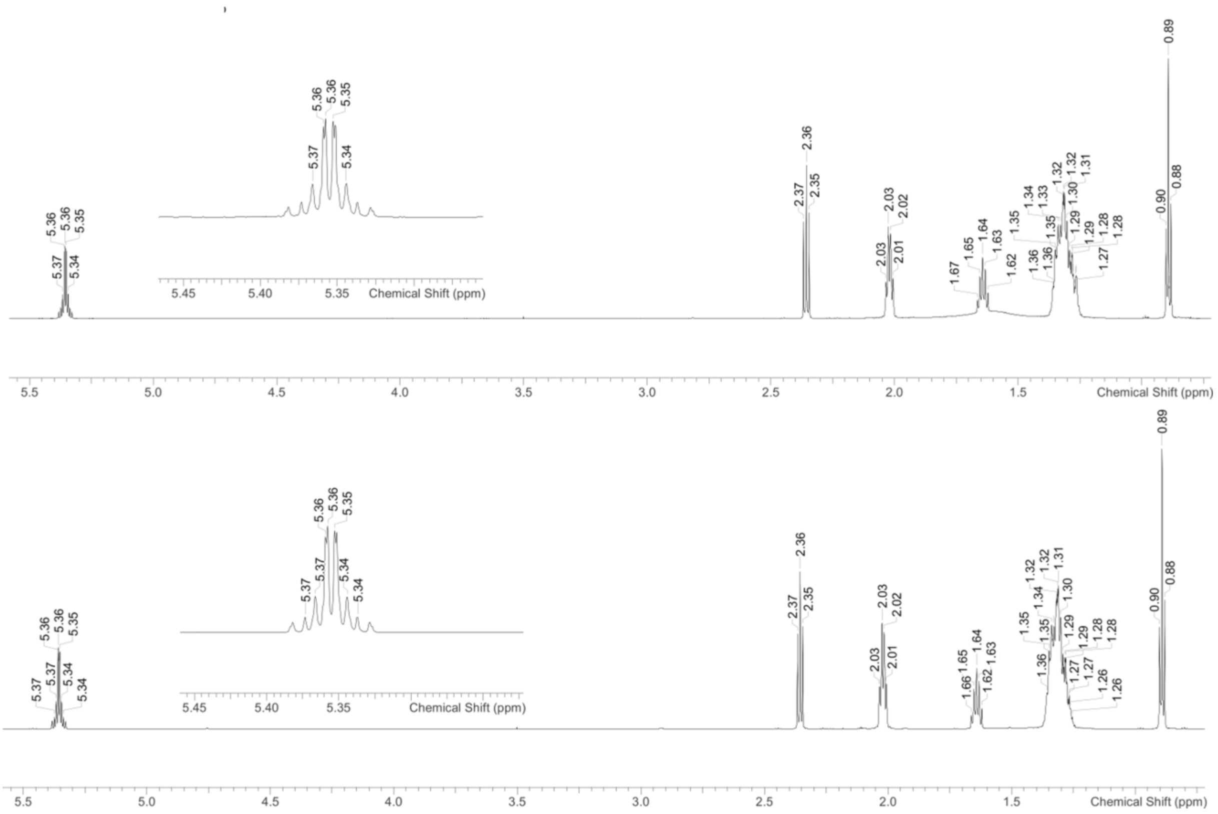


**Figure S2.** Comparison of the 700 MHz $^{1}$H NMR spectra of isolated (upper spectrum) and reference (lower spectrum) palmitoleic acid in $CDCl_3$.

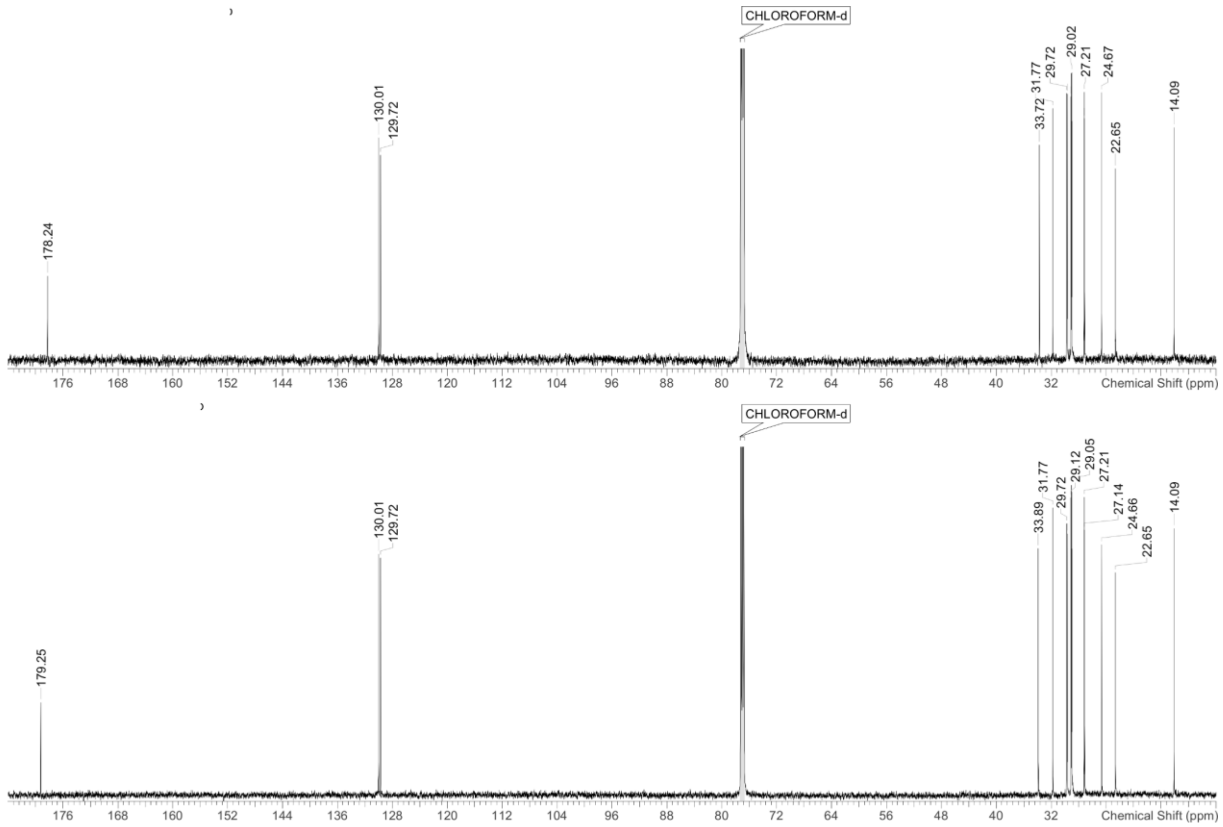


**Figure S3.** Comparison of the 176.1 MHz $^{13}$C NMR spectra of isolated (upper spectrum) and reference (lower spectrum) palmitoleic acid in $CDCl_3$.

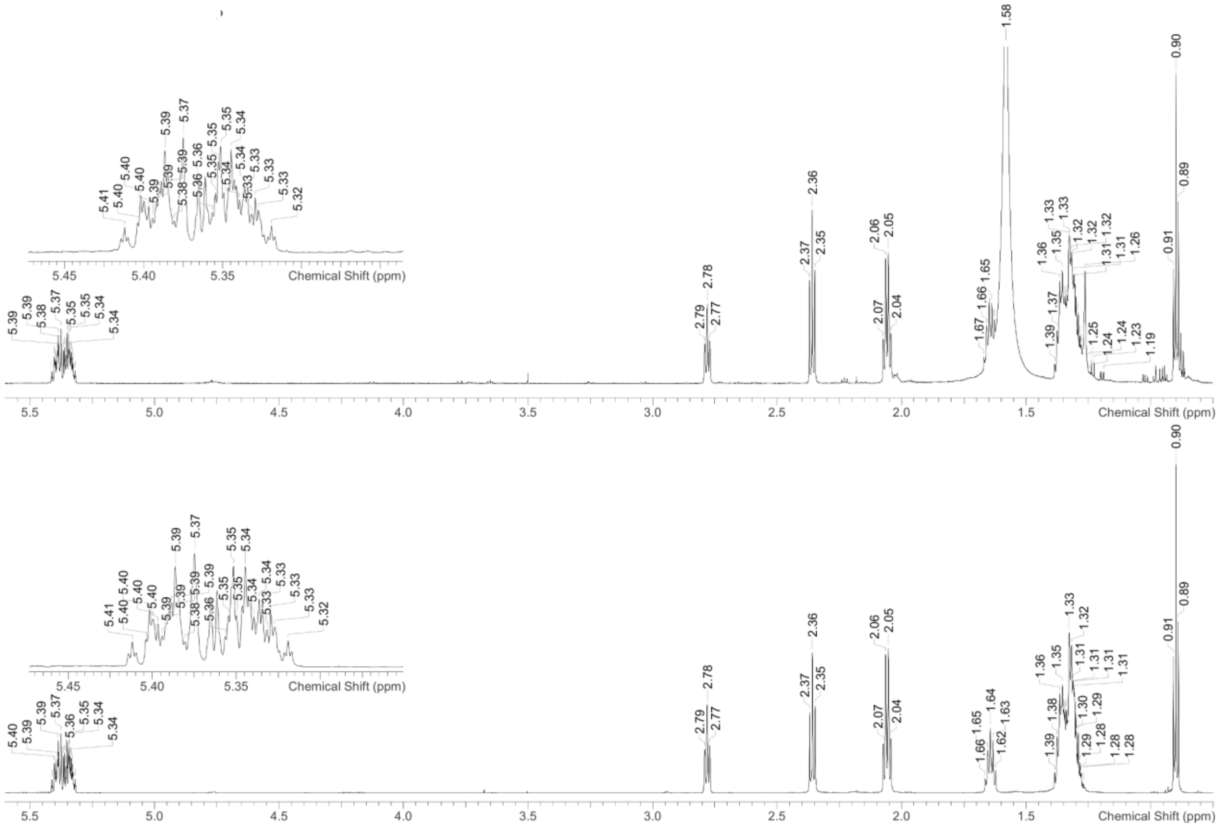


**Figure S4.** Comparison of the 700 MHz $^{1}H$ NMR spectra of isolated (upper spectrum) and reference (lower spectrum) linoleic acid in $CDCl_3$.

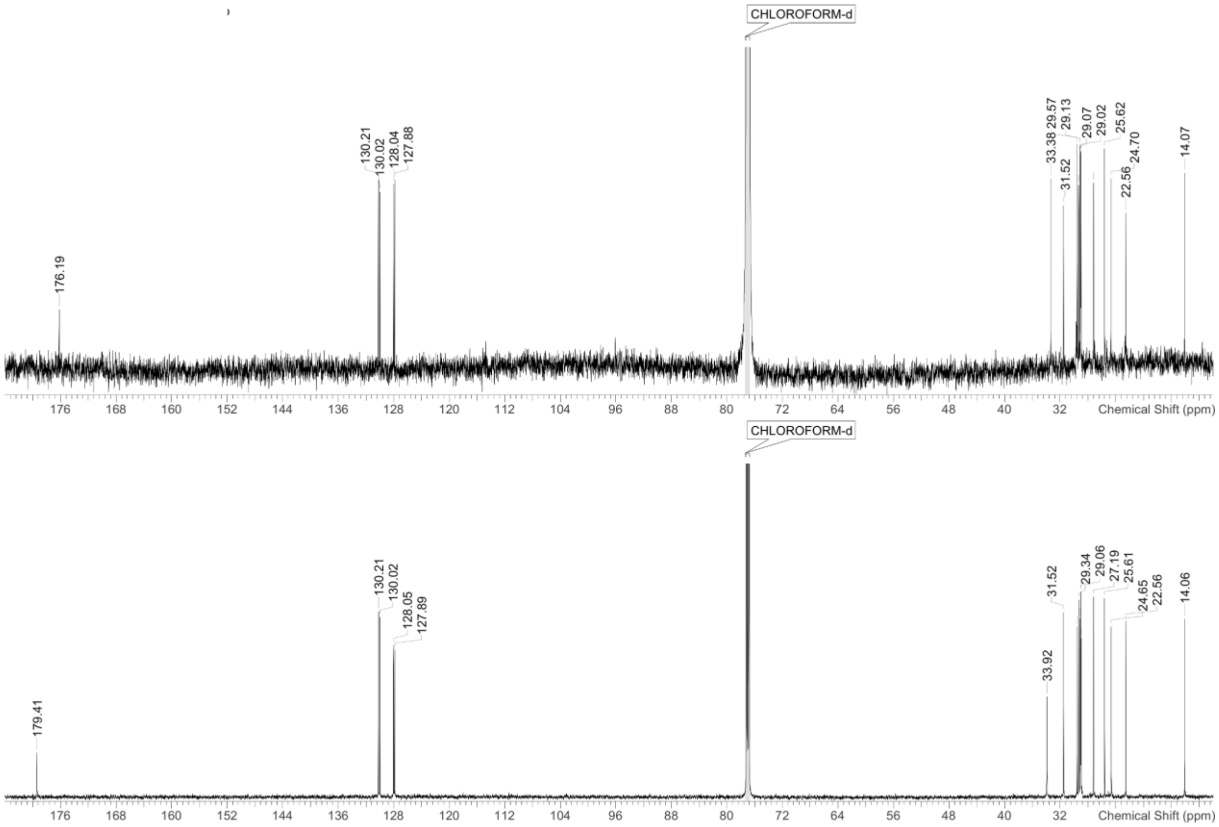


**Figure S5.** Comparison of the 176.1 MHz $^{13}C$ NMR spectra of isolated (upper spectrum) and reference (lower spectrum) palmitoleic acid in $CDCl_3$ (showing pH dependent shift differences).

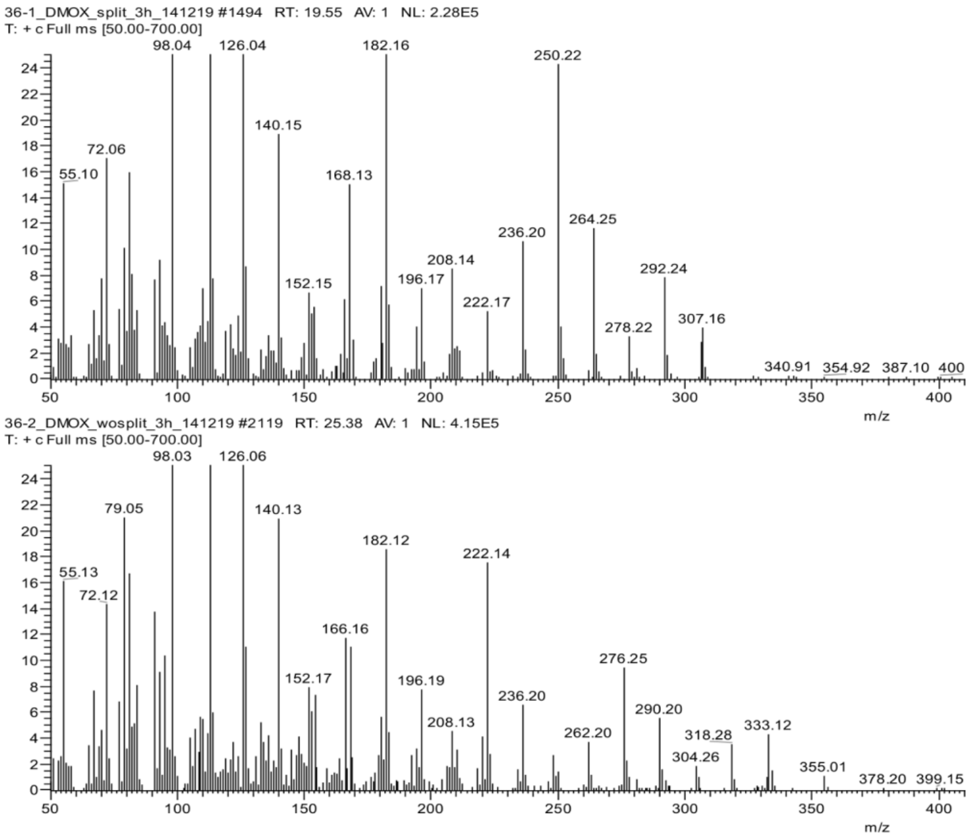


**Figure S6.** MS spectra of DMOX derivatives of RC 36.1 (upper spectrum) and RC 36.2 (lower spectrum). The molecular ion $[M+H]^+$ of the DMOX derivative of RC 36.1 is found at *m/z* 307. Characteristic fragments can be observed; fragmentation occurred at the site of the double bond or next to the double bond (*m/z* 196 and 208) indicating that the double bond is located at position 9 (upper spectrum). The molecular ion $[M+H]^+$ of the DMOX derivative of RC 36.2 was detected at *m/z* 333. In addition, characteristic fragments of the derivative of RC 36.2 were found at *m/z* 196, 208, 222, 238 and 262 indicating that the double bonds were located at positions 9 and 12 (lower spectrum).

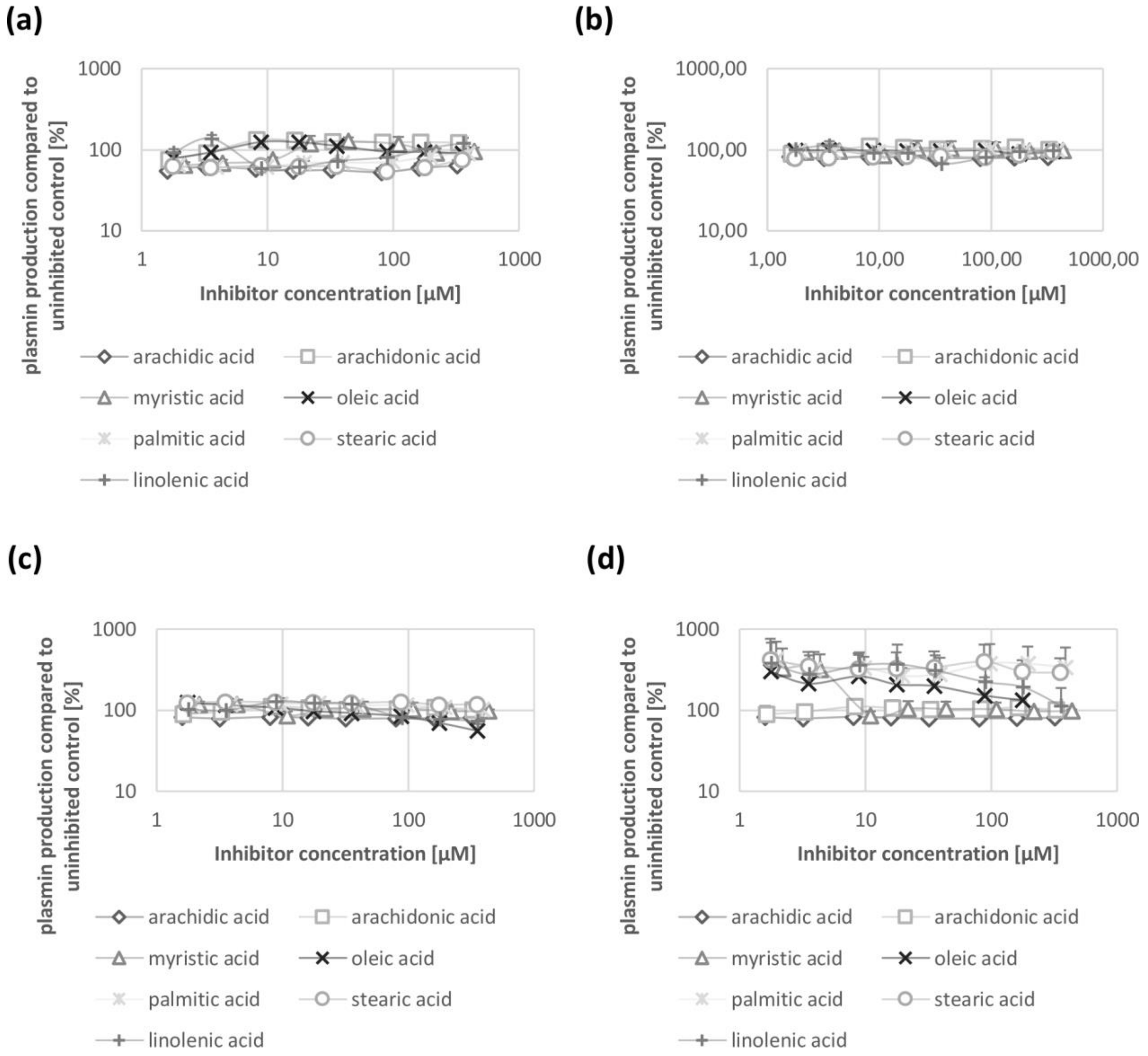


**Figure S7.** Arachidic, arachidonic, myristic, oleic, palmitic, stearic and linolenic acid were tested in cell-based plasminogen activation assays using cluster 2b type streptokinase (a) or cluster 2a type streptokinase (b). In addition, they were tested in supernatant-based plasminogen activation assays using cluster 2a type (c) or cluster 1 type streptokinase (d). Fatty acids do not show effects in cell-based plasminogen activation assays (a,b). In supernatant-based plasminogen activation assays with cluster 2a type streptokinase (c) only oleic and linolenic acid show slight inhibitory effects at high concentrations whereas these effects are not observed upon activation with cluster 1 type (d).

**Table S1.** NMR data of RC28.1 (RC 36.1) palmitoleic acid in $CDCl_3$[a]

| C | $\delta_C$ | m | H | $\delta_H$ | m | COSY | H in HMBC |
|---|---|---|---|---|---|---|---|
| 1 | 178.23 | C | | | | | 3, 2 |
| 2 | 33.72 | $CH_2$ | 2 | 2.36 | t[b] | 3 | 3 |
| 3 | 24.67 | $CH_2$ | 3 | 1.64 | dt[c] | 2, 4 | 2 |
| 4 | 29.13 | 6 | 4 | 1.33 | m | 3 | |
| 5 6 | 28.98 | $CH_2$ | 5 | 1.26 | m | | |
| 7 12 13 | 29.02 | | 6 | – | | | |
| | 29.05 | | 7 12 | 1.37 | | | |
| | 29.66 | | 13 | | | | |
| | 29.72 | | | | | | |
| 8 11 | 27.14 | 2 | 8 11 | 2.92 | m | | |
| | 27.21 | $CH_2$ | | | | | |
| 9 10 | 130.00 | 2 CH | 9 10 | 5.35 | m | | |
| | 129.71 | | | | | | |
| 14 | 31.77 | $CH_2$ | 14 | 1.28 | m | 16, 15 | 16, 15 |
| 15 | 22.65 | $CH_2$ | 15 | 1.30 | m | 16, 14 | 16, 14 |
| 16 | 14.10 | $CH_3$ | 16 | 0.89 | t[d] | 15, 14 | 15 |

[a] $^1H$ 700.4 MHz, $^{13}C$ 176.1 MHz; [b] 7.5 Hz; [c] 14.9 and 7.5 Hz; [d] 7.1 Hz

**Table S2.** NMR data of RC28.3 (RC 36.2) linoleic acid in $CDCl_3$.

| C | $\delta_C$ | m | $\delta_H$ | m | COSY | H in HMBC |
|---|---|---|---|---|---|---|
| 1 | 176.19 | C | | | | 3, 2 |
| 2 | 33.38 | $CH_2$ | 2.36 | t[b] | 3 | 3 |
| 3 | 24.70 | $CH_2$ | 1.65 | m | 4, 2 | 2 |
| 4 | 29.02 | 5 | 1.39 | m | | |
| 5 | 29.07 | $CH_2$ | - | | | |
| 6 | 29.13 | | 1.25 | | | |
| 7 15 | 29.34 | | | | | |
| | 29.57 | | | | | |
| 8 14 | 27.20 | 2 | 2.06 | q[c] | 15/7, | 12/10, 15/7 |
| | 27.17 | $CH_2$ | | | 11, 13/9 | |
| 11 | 25.62 | $CH_2$ | 2.78 | t[d] | 8/14, | 13/9 |
| | | | | | 12/10 | |
| 12 10 | 127.88 | 2 | 5.34 | m | 11, 13/9 | 8/14, 11, |
| | 128.04 | CH | | | | |
| 13 9 | 130.02 | 2 | 5.38 | m | 8/14, | 8/14, 11 >15/7 |
| | 130.21 | CH | | | 12/10 | |
| 16 | 31.52 | $CH_2$ | 1.30 | m | | 18, 17, 15, 14 |
| 17 | 22.56 | $CH_2$ | 1.31 | m | 18, 16 | 18, 16 |
| 18 | 14.07 | $CH_3$ | 0.90 | t[e] | 17 | 17 |

[a] $^1$H 700.4 MHz, $^{13}$C 176.1 MHz; [b] 7.5 Hz; [c] 7.1 Hz; [d] 7.0 Hz; [e] 7.1 Hz

**Table S3.** Primers and reaction conditions for amplification of *ska* gene.

| Primer | Sequence |
|---|---|
| **ska_M6_fwd (for A614)** | 5'-GCG GAT CCA TTG CTG GGT ATG GAT GG-'3 |
| **ska_M1M6_rev** | 5'-GCG TCG ACT TAT TTG TCT TTA GGG TTA TC-'3 |
| | |
| **Protocol for PCR amplification** | |
| Phusion Hot Start PCR buffer | 5 µl |
| magnesium chloride 50 mM | 2.5 µl |
| dNTPs | 2 µl |
| ska_M1M6_rev (10 pmol/µl) | 1 µl |
| ska_M6_fwd (for A614) | 1 µl |
| Phusion Polymerase | 0.25 µl |
| respective DNA sample | 2 µl |
| water | 11.25 µl |
| | |
| **Thermal cycling conditions (steps 2-4 were repeated 29 times)** | |
| (1) 98°C 30 sec | |
| (2) 98°C 10 sec | |
| (3) 65°C 30 sec | |
| (4) 72°C 80 sec | |
| (5) 72°C 7 min | |
| (6) 4 °C hold | |
